# Brain Mechanism of Acupuncture Treatment of Chronic Pain: An Individual-Level Positron Emission Tomography Study

**DOI:** 10.3389/fneur.2022.884770

**Published:** 2022-05-02

**Authors:** Jin Xu, Hongjun Xie, Liying Liu, Zhifu Shen, Lu Yang, Wei Wei, Xiaoli Guo, Fanrong Liang, Siyi Yu, Jie Yang

**Affiliations:** ^1^Department of Acupuncture and Tuina, Chengdu University of Traditional Chinese Medicine, Chengdu, China; ^2^Department of Nuclear Medicine, Sichuan Academy of Medical Sciences and Sichuan Provincial People's Hospital, Chengdu, China; ^3^Department of Traditional Chinese and Western Medicine, North Sichuan Medical College, Nanchong, China

**Keywords:** acupuncture, metabolic, biomarker, primary dysmenorrhea, machine learning

## Abstract

**Objective:**

Acupuncture has been shown to be effective in the treatment of chronic pain. However, their neural mechanism underlying the effective acupuncture response to chronic pain is still unclear. We investigated whether metabolic patterns in the pain matrix network might predict acupuncture therapy responses in patients with primary dysmenorrhea (PDM) using a machine-learning-based multivariate pattern analysis (MVPA) on positron emission tomography data (PET).

**Methods:**

Forty-two patients with PDM were selected and randomized into two groups: real acupuncture and sham acupuncture (three menstrual cycles). Brain metabolic data from the three special brain networks (the sensorimotor network (SMN), default mode network (DMN), and salience network (SN)) were extracted at the individual level by using PETSurfer in fluorine-18 fluorodeoxyglucose positron emission tomography (^18^F-FDG-PET) data. MVPA analysis based on metabolic network features was employed to predict the pain relief after treatment in the pooled group and real acupuncture treatment, separately.

**Results:**

Paired *t*-tests revealed significant alterations in pain intensity after real but not sham acupuncture treatment. Traditional mass-univariate correlations between brain metabolic and alterations in pain intensity were not significant. The MVPA results showed that the brain metabolic pattern in the DMN and SMN did predict the pain relief in the pooled group of patients with PDM (*R*^2^ = 0.25, *p* = 0.005). In addition, the metabolic pattern in the DMN could predict the pain relief after treatment in the real acupuncture treatment group (*R*^2^ = 0.40, *p* = 0.01).

**Conclusion:**

This study indicates that the individual-level metabolic patterns in DMN is associated with real acupuncture treatment response in chronic pain. The present findings advanced the knowledge of the brain mechanism of the acupuncture treatment in chronic pain.

## Introduction

Acupuncture, a traditional Chinese medical procedure, has been widely used to alleviate diverse pains for over 2,000 years (1). The National Institutes of Health have suggested acupuncture as a potentially useful option for various chronic pain disorders, such as menstrual pain (2). Primary dysmenorrhea (PDM), a classic chronic and cyclic pain disorder, is characterized by cyclic cramping pain in the lower abdomen during menstruation and a lack of any visible pelvic pathology. PDM affects most women throughout the menstrual years, with up to 90% of adolescent females globally reported experiencing it (3). Although PDM is a common reason for work absenteeism and lower quality of life in women, the disorder is often under-diagnosed and poorly treated (4). Several randomized controlled trials (RCTs) further suggest favorable effects of acupuncture on menstrual pain intensity and other symptoms of dysmenorrhea (5–7). Moreover, our previous meta-analysis also demonstrated that acupuncture is safe and effective in PDM management (8), and real acupuncture could be more effective than placebo/sham group in pain relief (9).

Although acupuncture has been shown to be effective in pain relief in patients with PDM, the inter-individual response of different patients to acupuncture treatment varies greatly (10–12). Similarity, responses to other treatments for pain are also be affected by multidimensional individual differences (13–15). Because pain is a highly personal and subjective experience, it is not surprising that the outcome of treatment is influenced by the baseline physiological state of individuals (16, 17). Thus, the baseline brain characteristics of individuals might be a useful biomarker to predict differential responses to intervention strategies.

Neuroimaging biomarkers have recently proven promising for predicting responses to treatment and are used for elucidating the underlying brain mechanism for pain relief. For example, Reggente et al. (18) found that pre-treatment brain connectivity in the visual network and the default mode network (DMN) significantly predicted obsessive-compulsive disorder severity after treatment. Conversely, clinical pre-treatment variables did not reliably predict post-treatment outcomes, indicating that brain networks are stronger predictors than more readily obtained clinical scores. A recent study found that resting-state regional homogeneity in the temporoparietal junction was an important predictive factor of treatment effects of acupuncture in patients with cervical spondylosis neck pain (19). As such earlier studies show successful applications of brain-based biomarkers to predict therapeutic effects at the individual level, the development of quantitative, objective neuroimaging biomarkers/predictors is of increasing importance to provide optimal treatment for PDM and provides a useful approach to illustrate the broad applicability of acupuncture. In the past few years, neuroimaging studies of PDM have increasingly and collectively shown that PDM is associated with significant changes in brain anatomy, function, and metabolism (20–24). However, no study investigates the individual-level metabolic biomarker of treatment response in PDM.

In this exploratory study, we thus aimed to investigate whether individual-level brain metabolic biomarkers in a special network at baseline can predict acupuncture responses in the treatment of PDM using multivariate pattern analysis (MVPA)-based machine-learning techniques. We first acquired metabolic data from 42 individuals with PDM who underwent fluorine-18 fluorodeoxyglucose positron emission tomography (^18^F-FDG PET-CT) at baseline. Participants were then randomized into a real group and a sham acupuncture group and treated over the course of three menstrual cycles. Lastly, MVPA was applied to explore the optimal metabolic predictors for clinical responses after treatment in subjects with PDM. To improve the analytic power and efficiency in this study, we restricted them to some special networks, such as the DMN, the sensorimotor network (SMN), and the salience network (SN), where previous studies found abnormal alterations in patients with chronic pain. We hypothesized that the individual-level metabolic patterns of these target networks can predict acupuncture responses in patients with PDM.

## Materials and Methods

### Participants

**Figure 1:**
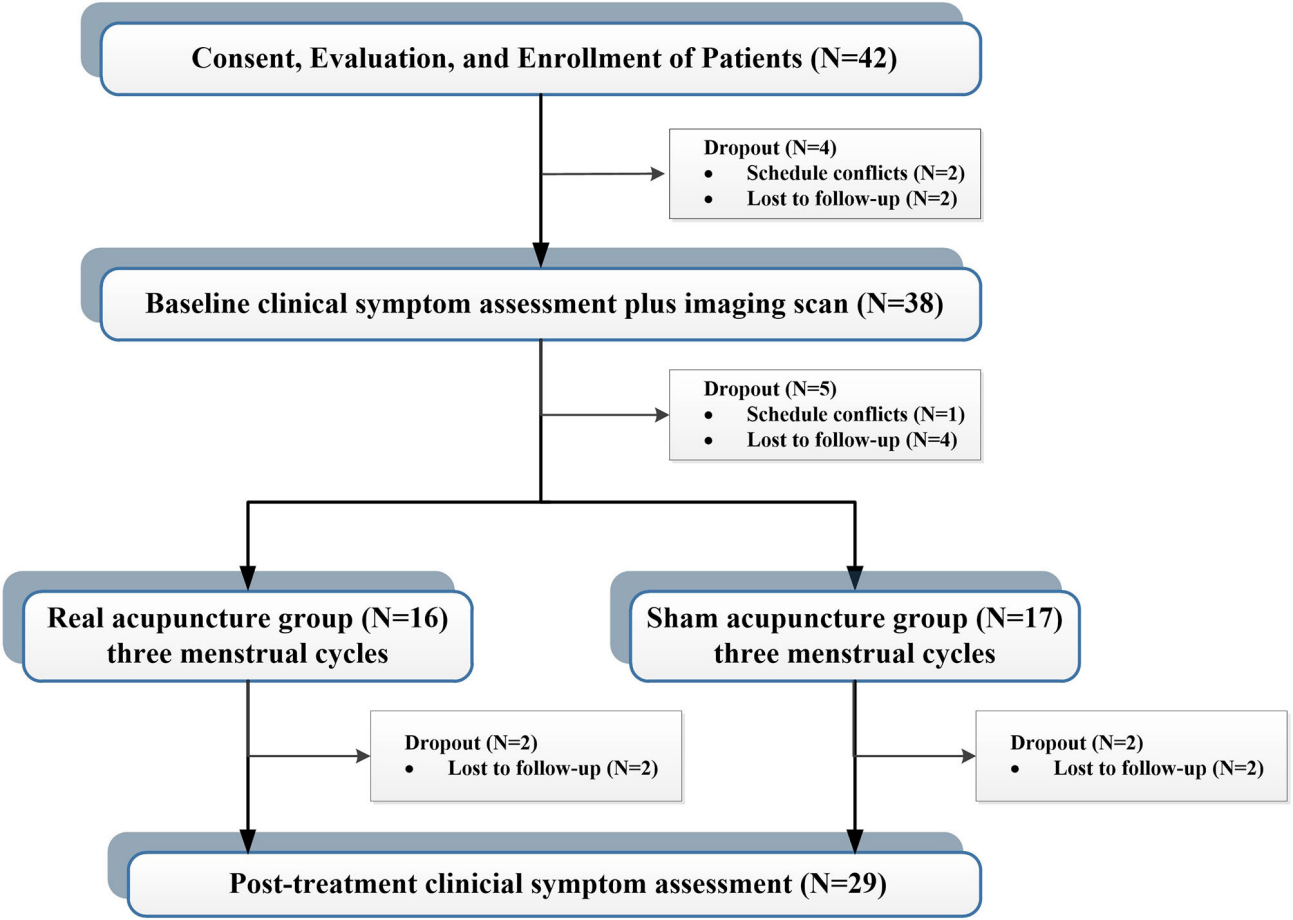
Study design and research flow chart.

A total of 42 patients with PDM were enrolled *via* advertisements and hearsay, and all participants were confirmed through telephone and face-to-face interviews. This study was approved by the affiliated Hospital of Chengdu University of Traditional Chinese Medicine Institutional Review Board. Before participation, all patients provided voluntary informed consent. The inclusion criteria for enrollment were fellows: (1) between the ages of 18 and 30; (2) a regular menstrual cycle (27–32 days); (3) more than 1 year of PDM history; (4) no hormones or centrally acting medication in the last 6 months; (5) cramping pain during menstruation at least 4 on a 0–10 visual analog scale (VAS); and (6) right-handedness. Exclusion criteria were as follows: (1) organic pelvic diseases; (2) visceral pain caused by other diseases; (3) a positive pregnancy test; (4) a history of neurological or psychiatric disorders; and (5) any contraindications to PET or MRI scanning. During the study, four cases were dropped out before the first clinical measurement and imaging scan, and five cases were dropped out before the first acupuncture treatment. Four cases did not complete all treatment sessions. Finally, 29 patients completed all clinical assessments and imaging sessions (Figure 1).

### Clinical Assessment

In this study, we used VAS (0 = “no pain at all,” 10 = “unbearable pain”) as primary outcome measurement to assess menstrual pain severity (25). In addition, the Zung Self-Rating Depression Scale (SDS) and the Zung Self-Rating Anxiety Scale (SAS) were also used to access the mental state of participants (26, 27). All clinical assessments were measured at baseline and after 3 menstrual cycles' acupuncture treatments.

### Acupuncture Intervention

Patients were randomly assigned to either the real or sham acupuncture groups using a computer-generated random-allocation process. All participants and clinical evaluators were unaware of the group assignment until the end of the study. Only the acupuncturists were informed about the treatment allocation and accordingly delivered real or sham treatment. Two licensed acupuncturists with at least 3 years of experience performed acupuncture on two groups of PDM patients. Participants received a course of acupuncture treatment that lasted 5–7 days before menstrual onset. The treatments lasted 3 weeks.

Based on the date-driven results of previous research and expert opinions, the *Sanyinjiao* (SP6) point was chosen for the real acupuncture treatment (28). The SP6 is located on the posterior border of the tibia and 3 *cun* directly superior to the tip of the medial malleolus (29). After the skin has been cleaned with 75% alcohol, 0.25 × 40-mm stainless needles (Hwatuo, Suzhou, China) were inserted to a depth of 1.0–1.2 *cun* and gently twisted, lifted, and thrust at an even amplitude, force, and speed. “Deqi” sensation of a soreness, numbness, heaviness, and distension sensation was essential during and after the operation. A 30-s manipulation was conducted every 10 min during 30-min needling retention. For the sham group, a nearby sham acupoint was chosen at the same level as the SP6 and *Xuanzhong* (GB39), at the midpoint of the stomach and gallbladder meridians. Patients in the sham group had an acupuncture technique comparable to those in the actual acupuncture group. However, there was no manipulation following needle insertion, and the “Deqi” sensation was not required.

### Statistical Analysis of Clinical Data

All statistical analyses were conducted in SPSS (SPSS statistical software, version 22.0, SPSS Inc., Chicago, IL, USA). The inner-group difference in VAS score (post-treatment minus pre-treatment) across each treatment group was tested by using paired t-test. Furthermore, a two-sample *t*-test has been used to compare the within-group difference of VAS score change (post-treatment minus pre-treatment) between the real and sham acupuncture groups. In addition, the relationships between before and after treatment clinical features were assessed with partial correlation analysis, controlling for age and treatment method. The significant level for all analyses was set at *p* < 0.05.

### Imaging Acquisition

The structural MRI (sMRI) data were acquired to co-register the brain region to the PET image. A 32-channel radio-frequency head coil in a 3.0-Tesla magnetic resonance scanner was used to collect MRI data (Discovery MR750, General Electric, Milwaukee, WI, USA). To limit head motion and scanner noise, earplugs and tight yet soft foam padding were used. The following parameters were used to create a high-resolution, T1-weighted structural image: repetition time = 2,530 ms, echo time = 3.39 ms, field of view = 256 mm × 256 mm, data matrix = 256 × 256; slice thickness = 1 mm, gap = 0 mm, and flip angle = 7°. Referring to our previous studies (30), ^18^F-FDG-PET scanning was prepared at Sichuan Provincial People's Hospital using a Biograph Duo BGO scanner (Siemens, Munich, Germany). The FDG-PET image was scanned in the morning during the periovulatory phase (the middle 5 days between the two menstrual periods). After fasting for at least 12 h, patients underwent the following procedures: (1) fasting plasma glucose and resting blood pressure measurements at 8 a.m., (2) a 15–20 min peaceful rest in a darkroom, (3) an intravenous injection of fluorine-18 fluorodeoxyglucose on the back of the right hand (synthesized with a Mini Tracer accelerator at 0.11 MCi/kg dosage), (4) a 40-min rest, and (5) a PET-CT scan. Before picture capture, there was a 40-min uptake period. Patients were encouraged to stay motionless during scanning by having their heads immobilized, their ears muffled, and their eyes blinded.

### Region of Interest (ROI) Selection

**Figure 2:**
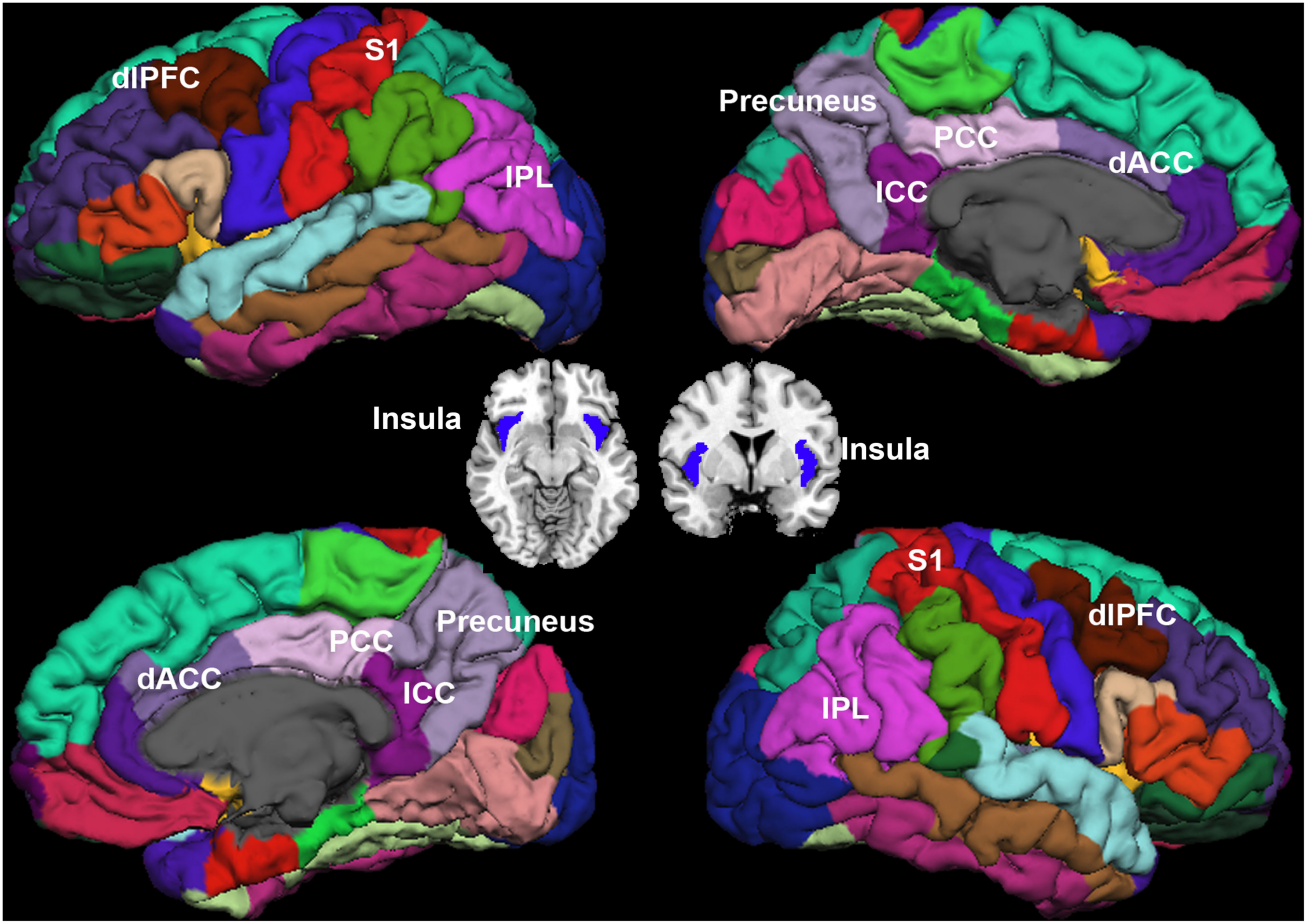
Regions of interest. Sensorimotor network (SMN): bilateral post-central (S1), and bilateral insula. Salience network (SN): the dorsolateral prefrontal cortex (dlPFC), and bilateral dorsal anterior cingulate cortex (dACC). Default mode network (DMN): bilateral inferior parietal cortex (IPC), precuneus, isthmus cingulate cortex (ICC), and posterior cingulate cortex (PCC).

Sixteen ROIs in the cortical region were selected for further analysis (Figure 2). Four regions were identified as key regions within the SMN, the bilateral post-central (S1), and bilateral insula (31, 32), which represent the major ascending pathways of pain; four regions were selected in the SN, which represent the descending pathways that modulate pain by inhibiting nociceptive transmission, such as the bilateral caudal middle frontal gyrus (a region within the dorsolateral prefrontal cortex, dlPFC), the bilateral caudal anterior cingulate cortex (a region in the dorsal anterior cingulate cortex, dACC) (33); and eight regions were selected in the DMN, which are involved in pain rumination (34), such as the bilateral inferior parietal cortex (IPC), the precuneus, the isthmus cingulate cortex, and the posterior cingulate cortex (PCC).

### Imaging Processing and Individual-Level Metabolic Extraction

The pre-processing of sMRI and metabolic data was conducted using FreeSurfer and PETSurfer toolbox (version 6.0, http://surfer.nmr.harvard.edu/). First, the cortical parcellation and subcortical segmentation methods have been described in detail in our prior publications (35, 36). Second, the standardized uptake value rate (SUVR) was calculated for the metabolic state in the brain regions. The SUVR is useful for normalizing the comparison of the significant inter-individual variability in the global PET signal (37). A symmetric geometric transfer matrix (SGTM) method was used for partial volume correction (PVC) where limited scanner resolution causes the activity to appear to spill out of one region and into another (38). To perform PVC, the PET data were registered to the MRI data *via* boundary-based registration (BBR) using a six degree of freedom linear transform (39). The MRI segmentation was mapped onto the PET space in a way that accounted for the tissue fraction effect (38). The SUVR for each ROI was computed by dividing the intensity of the ROI by the intensity of the pons at the individual level (39) (FreeSurfer commands: gemseg, mri_coreg, and mri_gtmpvc). Third, the SUVR data of the 16 ROIs were extracted from the processed images at the individual level for further analysis.

### Multivariate Pattern Analysis

**Figure 3:**
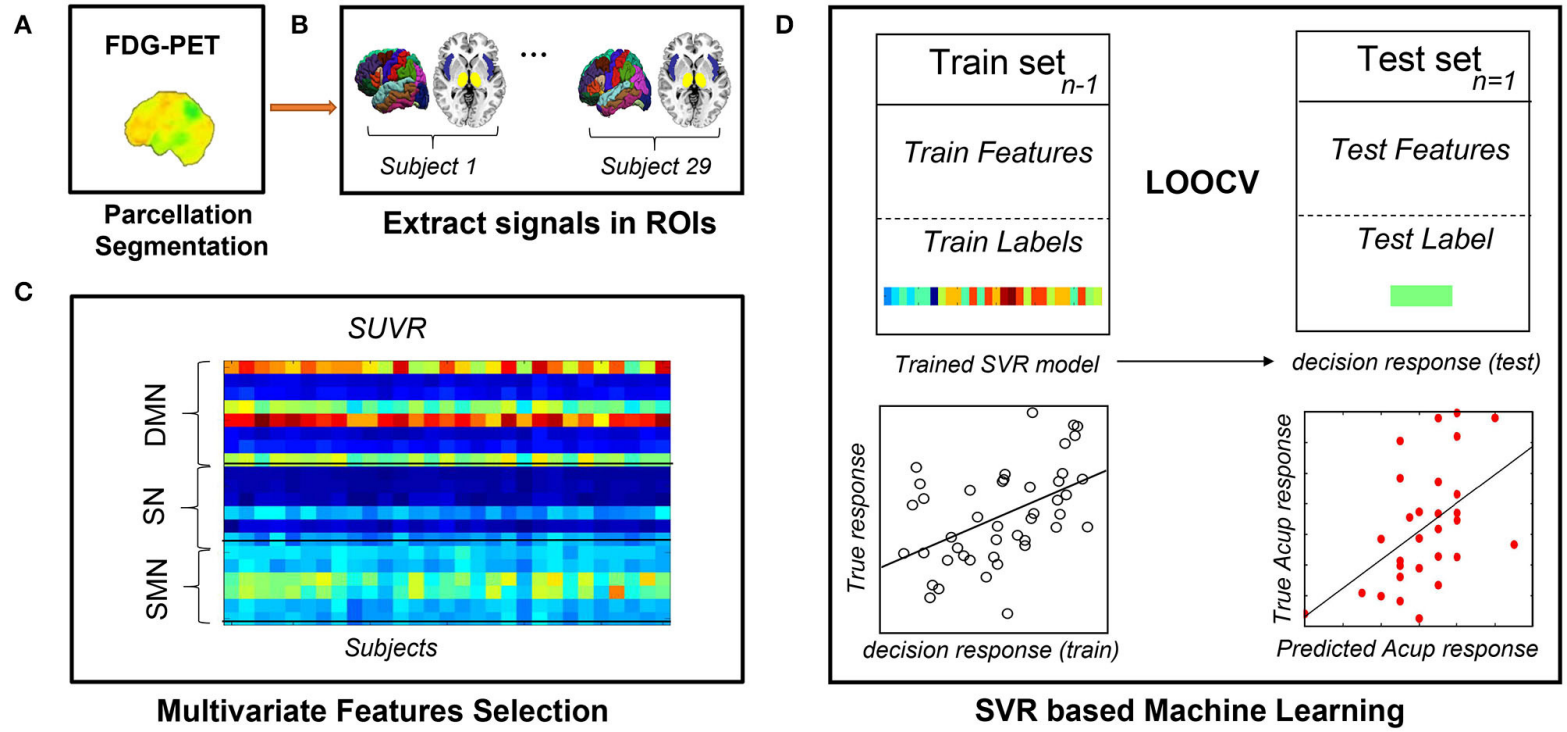
Flowchart of the MVPA procedure. **(A)** Obtaining quantitative information from preprocessed PDG-PET scans. **(B)** Extracting metabolism data across all voxels in all ROIs. **(C)** Constructing feature matrixes of the SUVR. **(D)** Building the SVR model with LOOCV to predict each participant's response to acupuncture. FDG-PET, fluorodeoxyglucose positron emission tomography; LOOCV, leave-one-out cross-validation; ROIs, regions of interest; sMRI, structural magnetic resonance imaging; SUVR, standardized uptake value ratio; SVR, support vector regression.

We attempted to predict clinical symptom alterations after the treatment based on special metabolic networks. We used linear support vector regression (SVR) that was implemented with the LIBSVM toolbox (40) for model training and further prediction analysis. We used the change in pain severity (VAS change) as the dependent variable and brain metabolic network features as independent variables (predictors) while controlling for effects of age, treatment method (only in the pooled group prediction analysis, see below), and VAS score at baseline. A leave-one-out cross-validation (LOOCV) method was used to ensure a clear separation between training and test sets. LOOCV is appropriate for preliminary estimate prediction in longitudinally neuroimaging studies where sample sizes are small (41). To evaluate the predictive ability of SVR, we calculated the mean absolute error, defined as the mean discrepancy between actual and predicted values (42, 43), and the squared prediction-outcome correlation (*R*^2^), defined as the squared correlation between the prediction and true outcome. Furthermore, we estimated the probability that random chance would predict the treatment response and SVR method (permutation test with 10,000 iterations). Because of the small sample size of the present study, we combined the real and sham groups for a pooled group MVPA analysis, where the treatment method effect was controlled as a covariate. In addition, the MVPA analysis was also conducted within each group separately. Figure 3 illustrates the individualized prediction framework used in this investigation.

### Mass-Univariate Correlation Analysis

To directly compare our multivariate SVR analysis with traditional mass-univariate correlation analyses, we tested the association between treatment responses and metabolic properties in each ROI using a traditional bivariate Pearson correlation analysis. The significance level was set at *p* < 0.05.

## Results

### Demographic Characteristics and Clinical Results

As shown in Table 1, statistical analysis indicats no significant difference in age, disease duration, body mass index (BMI), baseline VAS scores, SDS scores, and SAS scores between the two treatment groups at the baseline stage (all *p* > 0.05). For intra-group comparison, the post-treatment VAS value was significantly decreased in the real acupuncture group than baseline (*T* = 6.19, *p* = 3.28 × 10^−5^); however, no change was detected in the sham acupuncture group throughout the trial (*T* = 1.67, *p* = 0.12). For inter-group comparison, there was a significant difference in the real acupuncture vs. sham acupuncture group in the changes of VAS value after three menstrual cycles of treatment (*T* = −3.17, *p* = 0.004). Baseline VAS scores were not significantly associated with VAS changes after treatment after controlling for age and treatment methods in the pooled group analysis (*R* = −0.34, *p* = 0.07) and the real acupuncture group (*R* = −0.20, *p* = 0.51). In addition, treatment responses did not correlate with disease duration, SAS, or SDS (all *p* > 0.05). In addition, no significant difference was found in the SUVR in all selected regions between the two groups (all *p* > 0.05), see Table 2.

**Table 1:** Demographics and clinical characteristics of each group.

	**Real (*****n*** **=** **14)**		**Sham (*****n*** **=** **15)**		***T***	***P***
	**Mean**	**SD**	**Mean**	**SD**		
Age (years)	24.86	1.75	24.53	2.07	0.45	0.653
Duration (months)	90.57	36.31	97.40	34.09	−0.52	0.606
BMI	19.20	1.15	19.45	1.81	−0.44	0.664
Baseline VAS	6.07	1.07	6.00	1.20	0.17	0.867
Baseline SDS	39.68	7.28	43.42	10.07	−1.14	0.265
Baseline SAS	41.34	5.56	40.55	7.80	0.31	0.758
Post-treatment VAS	3.50	1.70	5.30	1.39	−3.14	0.004
Change VAS	−2.57	1.55	−0.70	1.62	−3.17	0.004

*BMI, body mass index; VAS, visual analog scale; SAS, Self-Rating Anxiety Scale; SDS, Self-Report Depression Scale*.

**Table 2:** The SUVR in all selected regions of each group.

**Brain Networks**	**Regions**	**Real (*****n*** **=** **14)**		**Sham (*****n*** **=** **15)**		***T***	***P***
		**Mean**	**SD**	**Mean**	**SD**		
Salience network	Left dACC	3.05	0.18	3.09	0.20	1.05	0.30
	Left dlPFC	2.75	0.13	2.74	0.10	0.20	0.84
	Right dACC	2.77	0.18	2.85	0.24	−1.03	0.31
	Right dlPFC	2.68	0.15	2.67	0.12	0.07	0.95
Default mode network	Left IPC	2.54	0.12	2.54	0.07	−0.22	0.83
	Left ICC	2.50	0.09	2.50	0.14	−0.10	0.92
	Left PCC	2.67	0.10	2.69	0.11	−0.45	0.66
	Left PCU	2.52	0.11	2.48	0.10	0.87	0.39
	Right IPC	2.58	0.10	2.55	0.09	0.72	0.48
	Right ICC	2.47	0.17	2.52	0.11	−0.98	0.34
	Right PCC	2.63	0.12	2.70	0.10	−1.73	0.10
	Right PCU	2.54	0.09	2.56	0.08	−0.48	0.64
Sensorimotor network	Left S1	2.17	0.08	2.17	0.09	0.03	0.98
	Left Insula	2.93	0.16	2.91	0.15	0.29	0.78
	Right S1	2.13	0.08	2.17	0.11	−0.95	0.35
	Right Insula	2.88	0.18	2.94	0.11	−0.94	0.35

*dACC, dorsal anterior cingulate cortex; dlPFC, dorsolateral prefrontal cortex; IPC, inferior parietal cortex; ICC, isthmus cingulate cortex; PCC, posterior cingulate cortex; PCU, precuneus; S1, postcentral*.

### MVPA Analysis Results

**Figure 4:**
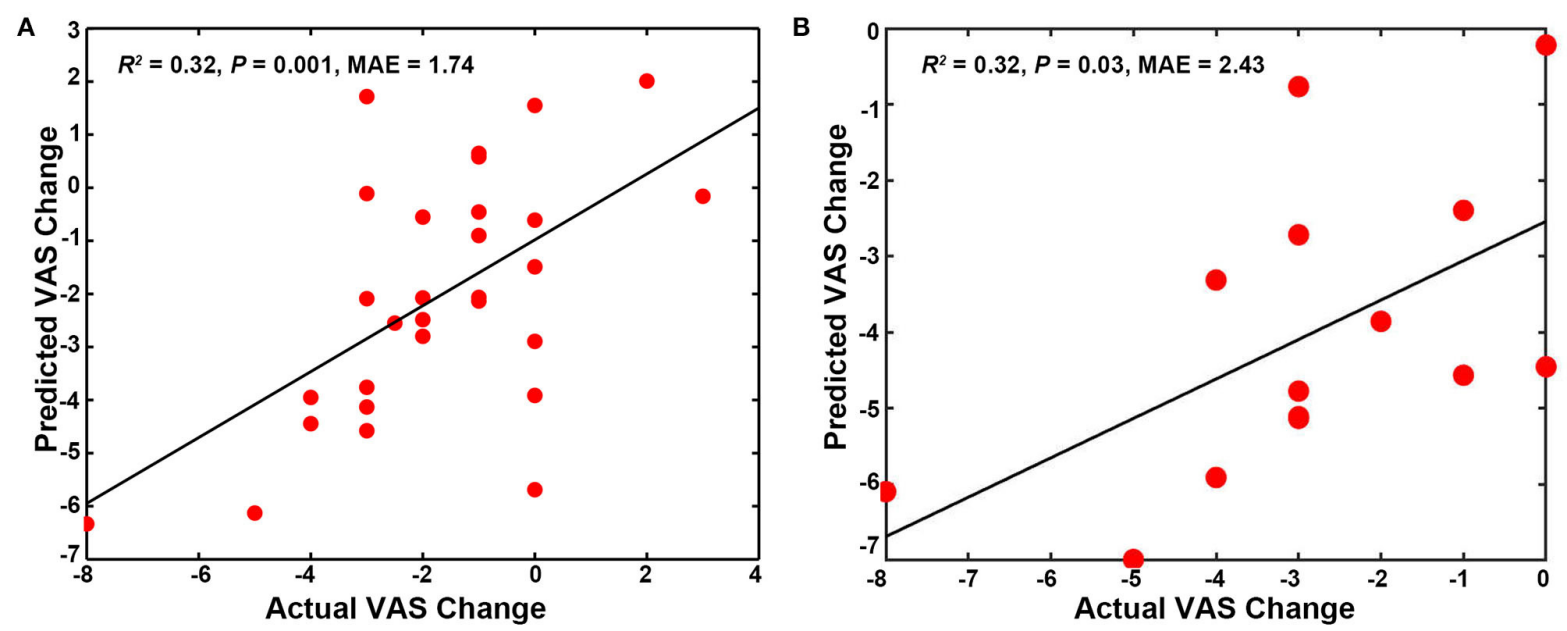
Predicting treatment effects using baseline SUVR patterns in special networks. **(A)** SUVR pattern in the SMN and DMN as a predictor for the pooled group. **(B)** SUVR pattern in the DMN as a predictor for the real acupuncture treatment group. DMN, default mode network; SMN, sensorimotor network; SN, salience network; SUVR, standardized uptake value ratio.

The SUVR patterns in the SMN and DMN could predict the VAS changes in the pooled group (SMN, *R*^2^ = 0.20, *p* = 0.01, Mean Absolute Error = 2.64; DMN, *R*^2^ = 0.14, *p* = 0.04, Mean Absolute Error = 2.51). In addition, the mixed SUVR pattern in the DMN and SMN could predict better for the VAS changes in the pooled group (*R*^2^ = 0.25, *p* = 0.005, Mean Absolute Error = 2.70; Figure 4A). In the real acupuncture group, the SUVR pattern in the DMN could predict the VAS changes after treatment (*R*^2^ = 0.40, *p* = 0.01, Mean Absolute Error = 2.71; Figure 4B). No other significant predictor was found in the real acupuncture or the sham acupuncture group.

### Mass-Univariate Correlation Results

To directly compare the multivariate pattern machine-learning analysis with traditional mass-univariate correlation analyses, we conducted Pearson correlation analyses. The results are displayed in Table 3: no significant association between regional SUVR and VAS changes is found using univariate correlation analysis.

**Table 3:** The relationship between change of VAS and brain features by using bivariate Pearson correlation analyses.

**Brain network**	**ROI**	***R***	***P***
Default mode network	Left IPC	0.29	0.12
	Left ICC	0.26	0.18
	Left PCC	0.25	0.19
	Left PCU	0.36	0.05
	Right IPC	0.30	0.11
	Right ICC	0.37	0.05
	Right PCC	0.29	0.13
	Right PCU	0.37	0.05
Salience network	Left dACC	0.14	0.48
	Left dlPFC	0.24	0.20
	Right dACC	0.30	0.11
	Right dlPFC	0.29	0.13
Sensorimotor network	Left S1	0.29	0.13
	Left Insula	0.34	0.08
	Right S1	0.32	0.09
	Right Insula	0.32	0.09

*dACC, dorsal anterior cingulate cortex; dlPFC, dorsolateral prefrontal cortex; IPC, inferior parietal cortex; ICC, isthmus cingulate cortex; PCC, posterior cingulate cortex; PCU, precuneus; S1, postcentral*.

## Discussion

To our knowledge, this is the first study using a brain metabolic biomarker to predict the pain relief after acupuncture treatment in chronic pain disorders. MVPA-based machine-learning approach was employed to explore whether pre-treatment brain metabolism in three special networks (SMN, DMN, and SN) can predict acupuncture treatment responses in patients with PDM. The MVPA results show that mixed DMN and SMN did indeed predict the observed pain relief in patients with PDM. Specially, the DMN metabolic pattern could predict the pain relief after real acupuncture treatment in patients with PDM. These findings support the brain metabolic mechanism in the acupuncture treatment for chronic pain.

Multivariate pattern analysis is a widely used machine-learning approach in the neuroimaging research field (44). This technique has been used to investigate the pathophysiology of chronic pain conditions, such as neck pain (19), trigeminal neuralgia (45), and chronic back pain (46). The present findings illustrated that metabolic features at baseline may be a useful predictor for acupuncture treatment responses in patients with PDM. In our study, the predictive power and strength of MVPA approaches were validated in two ways. First, we found that baseline clinical or demographic features were not enough to predict outcome responses. Our results converge with previous findings of more accurate predictions from neural than readily obtained clinical information (47, 48). The MVPA analysis, where baseline VAS scores were controlled as covariates, suggests that baseline biomarkers reflect the capacity of an individual with PDM to return to normalcy (quantified by their VAS score) after acupuncture, independent of their starting symptom severity. Second, the traditional mass-univariate correlations between brain metabolic features and VAS changes were not significant. MVPA technology can increase sensitivity to subtle and spatially distributed brain differences, which may not be detected by traditional approaches (49, 50). We thus demonstrate the feasibility and reliability of MVPA models for predicting clinical symptom changes after acupuncture treatment in patients with PDM.

In our SVR models, the multivariate pattern in the SMN and DMN significantly predicted the VAS changes. The key role of the DMN and SMN in predicting responses to acupuncture is consistent with the central role of these networks in the pathophysiology of PDM. In some previous studies, brain abnormalities in areas associated with DMN and SMN have been confirmed to be related to PDM. For instance, using PET (20) and sMRI (21, 51), Tu et al. found increases in metabolism and altered gray matter volumes in brain regions within the DMN in patients with PDM vs. healthy controls. Of note, the SMN is involved in the sensory-discriminative aspects of pain. A resting-state functional MRI (fMRI) study observed a trait-related reduction of functional connectivity between the DMN and the SMN during a pain-free phase, indicating that the altered DMN and SMN may be an ongoing representation of cumulative menstrual pain (52). Additionally, multiple neuroimaging studies have suggested that acupuncture may have analgesic effects by modulating the DMN and SMN (53–56), indicating that these brain networks also play an important role in mediating acupuncture effects. For example, Dhond et al. (57) demonstrated that acupuncture treatment induced hyperconnectivity of the DMN to pain, memory, and affective regions and also increased SMN connectivity to pain-related brain regions. Specially, for the real acupuncture treatment group, we also found that the metabolic in DMN, not SMN and SN, could predict the treatment response in PDM. The DMNs have been considered to be involved in pain rumination (34), and the structural, metabolic, and functional alterations in DMN have been manifested in amount of previous neuroimaging studies (21, 51). Taken together, these findings proposed that metabolic features within the DMN and SMN can be used not only to identify the pathogenesis of PDM but also to predict therapy responsiveness, especially, the DMN metabolic feature could predict the real acupuncture response for PDM.

It should be emphasized that we combined the two therapy groups into a pooled group to increase statistical power in the MVPA analysis. Although we have controlled for the treatment method as a covariate, the different neural mechanisms underlying the effects observed in the real and sham acupuncture groups might have influenced the present results (10). Our analyses also identify the MVPA model as a potential predictor for treatment responses in the real acupuncture but not the sham acupuncture group. Additional studies with larger sample sizes are needed to examine further predictors for real and sham treatments in PDM. In summary, our research is in line with the growing interest in multivariate neuroimaging features and machine-learning methods for therapeutic outcome prediction and the tailoring of personalized interventions (58–60). In recent years, machine-learning-based predictive models have been successfully applied in a variety of therapies, such as transcranial magnetic stimulation (61), electroconvulsive therapy (62), and vagus nerve stimulation (63). These studies are beginning to unlock the potential and value of machine learning in the clinical practice.

It is necessary to mention the study's limitations. First, our sample size was small, and we only recruited through one single site. As the sample size is small, the cross-validation method used here may cause some instability and biased estimates. As a result, our findings should be interpreted with caution. Larger sample sizes and multiple site data are needed to verify the findings in future studies. Second, the present study used the Sanyinjiao (SP6) point for acupuncture treatment. Our previous study has manifested that the acupoint has a specific neural response (64), thus, further studies need to investigate the acupuncture neural effect with other acupoints. Third, the present study did not explore the metabolic in brain stem regions, such as the ventral tegmental area (VTA) and periaqueductal gray (PAG), as the FreeSurfer does not segment these brain stem regions. The VTA and PAG have been shown to play important roles in chronic pain and are modulated by acupuncture treatment of chronic pain (65). Future studies should include these brain regions to get a comprehensive metabolic mechanism knowledge of chronic pain and acupuncture treatment.

## Conclusion

The present study shows that individual-level metabolic patterns of the DMN and SMN can predict the pain relief after acupuncture treatment for PDM. This preliminary study supports the potential of metabolic biomarkers and MVPA to predict therapeutic outcomes in patients with PDM. The present findings advanced the brain metabolic mechanism of the chronic pain treatment.

## Data Availability Statement

The raw data supporting the conclusions of this article will be made available by the authors, without undue reservation.

## Ethics Statement

The studies involving human participants were reviewed and approved by the affiliated Hospital of Chengdu University of Traditional Chinese Medicine Institutional Review Board. The patients/participants provided their written informed consent to participate in this study.

## Author Contributions

JY and FL supervised the project. LL, ZS, WW, and XG enrolled the participants and acquired the data. HX performed the PET scan. JY and JX administered and conceptualized the project. SY analyzed the data. JX and SY wrote and revised the manuscript. All authors read and approved the final manuscript.

## Funding

This work was supported by the programs of the National Natural Science Foundation of China (Nos. 81973966 and 81774440), China National Postdoctoral Program for InnovativeTalents (No. BX20190046), the Science and Technology Support Program of Nanchong (19SXHZ0100), the Doctoral Scientific Research Foundation of North Sichuan Medical College (CBY19-QD10), and Chengdu University of Traditional Chinese Medicine Xinglin Scholar Discipline Talent Research and Improvement Plan (BSH2019011).

## Conflict of Interest

The authors declare that the research was conducted in the absence of any commercial or financial relationships that could be construed as a potential conflict of interest.

## Publisher's Note

All claims expressed in this article are solely those of the authors and do not necessarily represent those of their affiliated organizations, or those of the publisher, the editors and the reviewers. Any product that may be evaluated in this article, or claim that may be made by its manufacturer, is not guaranteed or endorsed by the publisher.

## References

[1] Zhao ZQ. Neural mechanism underlying acupuncture analgesia. Prog Neurobiol. (2008) 85:355–75. 10.1016/j.pneurobio.2008.05.004 18582529

[2] Campbell MA McGrath PJ. Non-pharmacologic strategies used by adolescents for the management of menstrual discomfort. Clin J Pain. (1999) 15:313–20. 10.1097/00002508-199912000-00008 10617260

[3] Coco AS. Primary dysmenorrhea. Am Fam Physician. (1999) 60:489–96. 10465224

[4] O'Connell K Davis AR Westhoff C. Self-treatment patterns among adolescent girls with dysmenorrhea. J Pediatr Adolesc Gynecol. (2006) 19:285–9. 10.1016/j.jpag.2006.05.004 16873033

[5] Armour M Dahlen HG Zhu X Farquhar C Smith CA. The role of treatment timing and mode of stimulation in the treatment of primary dysmenorrhea with acupuncture: An exploratory randomised controlled trial. PLoS ONE. (2017) 12:e0180177. 10.1371/journal.pone.0180177 28700680 PMC5507497

[6] Ma YX Ye XN Liu CZ Cai PY Li ZF Du DQ. A clinical trial of acupuncture about time-varying treatment and points selection in primary dysmenorrhea. J Ethnopharmacol. (2013) 148:498–504. 10.1016/j.jep.2013.04.045 23684618

[7] Witt CM Reinhold T Brinkhaus B Roll S Jena S Willich SN. Acupuncture in patients with dysmenorrhea: a randomized study on clinical effectiveness and cost-effectiveness in usual care. Am J Obstet Gynecol. (2008) 198:166.e1–8. 10.1016/j.ajog.2007.07.041 18226614

[8] Yu SY Lv ZT Zhang Q Yang S Wu X Hu YP. Electroacupuncture is beneficial for primary dysmenorrhea: the evidence from meta-analysis of randomized controlled trials. Evid Based Complement Altern Med. (2017) 2017:1791258. 10.1155/2017/1791258 29358960 PMC5735637

[9] Smith CA Zhu X He L Song J. Acupuncture for primary dysmenorrhoea. Cochrane Database Syst Rev. (2011) Cd007854. 10.1002/14651858.CD007854.pub2 21249697

[10] Tu Y Ortiz A Gollub RL Cao J Gerber J Lang C. Multivariate resting-state functional connectivity predicts responses to real and sham acupuncture treatment in chronic low back pain. Neuroimage Clin. (2019) 23:101885. 10.1016/j.nicl.2019.101885 31176295 PMC6551557

[11] Liu J Mu J Chen T Zhang M Tian J. White matter tract microstructure of the mPFC-amygdala predicts interindividual differences in placebo response related to treatment in migraine patients. Hum Brain Mapp. (2019) 40:284–92. 10.1002/hbm.24372 30256491 PMC6865394

[12] Liu J Mu J Liu Q Dun W Zhang M Tian J. Brain structural properties predict psychologically mediated hypoalgesia in an 8-week sham acupuncture treatment for migraine. Hum Brain Mapp. (2017) 38:4386–97. 10.1002/hbm.23667 28608601 PMC6866832

[13] Angst MS Phillips NG Drover DR Tingle M Ray A Swan GE. Pain sensitivity and opioid analgesia: a pharmacogenomic twin study. Pain. (2012) 153:1397–409. 10.1016/j.pain.2012.02.022 22444188 PMC3377769

[14] Coghill RC Eisenach J. Individual differences in pain sensitivity: implications for treatment decisions. Anesthesiology. (2003) 98:1312–4. 10.1097/00000542-200306000-00003 12766637

[15] Wager TD Atlas LY Leotti LA Rilling JK. Predicting individual differences in placebo analgesia: contributions of brain activity during anticipation and pain experience. J Neurosci. (2011) 31:439–52. 10.1523/JNEUROSCI.3420-10.2011 21228154 PMC3735131

[16] Wandner LD Scipio CD Hirsh AT Torres CA Robinson ME. The perception of pain in others: how gender, race, and age influence pain expectations. J Pain. (2012) 13:220–7. 10.1016/j.jpain.2011.10.014 22225969 PMC3294006

[17] Fillingim RB. Individual differences in pain: understanding the mosaic that makes pain personal. Pain. (2017) 158 (Suppl 1):S11. 10.1097/j.pain.0000000000000775 27902569 PMC5350021

[18] Reggente N Moody TD Morfini F Sheen C Rissman J O'Neill J. Multivariate resting-state functional connectivity predicts response to cognitive behavioral therapy in obsessive-compulsive disorder. Proc Natl Acad Sci U S A. (2018) 115:2222–7. 10.1073/pnas.1716686115 29440404 PMC5834692

[19] Chen J Wang Z Tu Y Liu X Jorgenson K Ye G. Regional Homogeneity and multivariate pattern analysis of cervical spondylosis neck pain and the modulation effect of treatment. Front Neurosci. (2018) 12:900. 10.3389/fnins.2018.00900 30574062 PMC6292425

[20] Tu CH Niddam DM Chao HT Liu RS Hwang RJ Yeh TC. Abnormal cerebral metabolism during menstrual pain in primary dysmenorrhea. Neuroimage. (2009) 47:28–35. 10.1016/j.neuroimage.2009.03.080 19362153

[21] Tu CH Niddam DM Chao HT Chen LF Chen YS Wu YT. Brain morphological changes associated with cyclic menstrual pain. Pain. (2010) 150:462–8. 10.1016/j.pain.2010.05.026 20705214

[22] Zhang Q Yu S Wang Y Wang M Yang Y Wei W. Abnormal reward system network in primary dysmenorrhea. Mol Pain. (2019) 15:1744806919862096. 10.1177/1744806919862096 31286840 PMC6616063

[23] Chen T Mu J Xue Q Yang L Dun W Zhang M. Whole-brain structural magnetic resonance imaging–based classification of primary dysmenorrhea in pain-free phase: a machine learning study. Pain. (2019) 160:734–41. 10.1097/j.pain.0000000000001428 30376532

[24] Liu P Liu Y Wang G Li R Wei Y Fan Y. Changes of functional connectivity of the anterior cingulate cortex in women with primary dysmenorrhea. Brain Imaging Behav. (2018) 12:710–7. 10.1007/s11682-017-9730-y 28516336

[25] Larroy C. Comparing visual-analog and numeric scales for assessing menstrual pain. Behav Med. (2002) 27:179–81. 10.1080/08964280209596043 12165972

[26] Zung WW. A rating instrument for anxiety disorders. Psychosomatics. (1971) 12:371–9. 10.1016/S0033-3182(71)71479-0 5172928

[27] Zung WWK Richards CB Short MJ. Self-Rating Depression Scale in an Outpatient Clinic: Further Validation of the SDS. Arch Gen Psychiatry. (1965) 13:508–15. 10.1001/archpsyc.1965.01730060026004 4378854

[28] Yu S Yang J Yang M Gao Y Chen J Ren Y. Application of acupoints and meridians for the treatment of primary dysmenorrhea: a data mining-based literature study. Evid Based Complemen Altern Med. (2015) 2015:752194. 10.1155/2015/752194 25802545 PMC4354715

[29] World Health Organization Western Pacific Regional Office. WHO Standard Acupuncture Point Locations in the Western Pacific Region. Geneva: World Health Organization (2008).

[30] Zeng F Qin W Liang F Liu J Tang Y Liu X. Abnormal resting brain activity in patients with functional dyspepsia is related to symptom severity. Gastroenterology. (2011) 141:499–506. 10.1053/j.gastro.2011.05.003 21684280

[31] Tracey I Mantyh PW. The cerebral signature for pain perception and its modulation. Neuron. (2007) 55:377–91. 10.1016/j.neuron.2007.07.012 17678852

[32] Davis KD Flor H Greely HT Iannetti GD Mackey S Ploner M. Brain imaging tests for chronic pain: medical, legal and ethical issues and recommendations. Nat Rev Neurol. (2017) 13:624. 10.1038/nrneurol.2017.122 28884750

[33] Hemington KS Wu Q Kucyi A Inman RD Davis KD. Abnormal cross-network functional connectivity in chronic pain and its association with clinical symptoms. Brain Struct Func. (2016) 221:4203–19. 10.1007/s00429-015-1161-1 26669874

[34] Kumbhare DA Elzibak AH Noseworthy MD. Evaluation of Chronic Pain Using Magnetic Resonance (MR) Neuroimaging Approaches. Clin J Pain. (2017) 33:281–90. 10.1097/AJP.0000000000000415 27518493

[35] Li X Chen H Lv Y Chao HH Gong L Li CSR. Diminished gray matter density mediates chemotherapy dosage-related cognitive impairment in breast cancer patients. Sci Rep. (2018) 8:13801. 10.1038/s41598-018-32257-w 30218006 PMC6138678

[36] Yu S Shen Z Lai R Feng F Guo B Wang Z. The orbitofrontal cortex gray matter is associated with the interaction between insomnia and depression. Front Psychiatry. (2018) 9:651. 10.3389/fpsyt.2018.00651 30564152 PMC6288475

[37] Alshikho MJ Zürcher NR Loggia ML Cernasov P Chonde DB Garcia DI. Glial activation colocalizes with structural abnormalities in amyotrophic lateral sclerosis. Neurology. (2016) 87:2554–61. 10.1212/WNL.0000000000003427 27837005 PMC5207001

[38] Erlandsson K Buvat I Pretorius PH Thomas BA Hutton BF. A review of partial volume correction techniques for emission tomography and their applications in neurology, cardiology and oncology. Phys Med Biol. (2012) 57:R119. 10.1088/0031-9155/57/21/R119 23073343

[39] Greve DN Salat DH Bowen SL Izquierdo-Garcia D Schultz AP Catana C. Different partial volume correction methods lead to different conclusions: an 18F-FDG-PET study of aging. Neuroimage. (2016) 132:334–43. 10.1016/j.neuroimage.2016.02.042 26915497 PMC4851886

[40] Chang CC. Lin CJ. LIBSVM: A library for support vector machines. (2011) 2:1–27. 10.1145/1961189.1961199

[41] Plitt M Barnes KA Wallace GL Kenworthy L Martin A. Resting-state functional connectivity predicts longitudinal change in autistic traits and adaptive functioning in autism. Proc Natl Acad Sci U S A. (2015) 112:E6699–706. 10.1073/pnas.1510098112 26627261 PMC4672806

[42] Wager TD Atlas LY Lindquist MA Roy M Woo CW Kross E. An fMRI-based neurologic signature of physical pain. N Engl J Med. (2013) 368:1388–97. 10.1056/NEJMoa1204471 23574118 PMC3691100

[43] Lindquist MA Krishnan A Lopez-Sola M Jepma M Woo CW Koban L. Group-regularized individual prediction: theory and application to pain. Neuroimage, (2017) 145(Pt B):274–87. 10.1016/j.neuroimage.2015.10.074 26592808 PMC5071107

[44] Yang Z Fang F Weng X. Recent developments in multivariate pattern analysis for functional MRI. Neurosci Bull. (2012) 28:399–408. 10.1007/s12264-012-1253-3 22833038 PMC5561894

[45] Zhong J Chen DQ Hung PSP Hayes DJ Liang KE Davis KD. Multivariate pattern classification of brain white matter connectivity predicts classic trigeminal neuralgia. Pain. (2018) 159:2076–87. 10.1097/j.pain.0000000000001312 29905649

[46] Tu Y Jung M Gollub RL Napadow V Gerber J Ortiz A. Abnormal medial prefrontal cortex functional connectivity and its association with clinical symptoms in chronic low back pain. Pain. (2019) 160:1308–18. 10.1097/j.pain.0000000000001507 31107712 PMC6530583

[47] Frick A Engman J Wahlstedt K Gingnell M Fredrikson M Furmark T. Anterior cingulate cortex activity as a candidate biomarker for treatment selection in social anxiety disorder. BJPsych Open. (2018) 4:157–9. 10.1192/bjo.2018.15 29922481 PMC6003252

[48] Whitfield-Gabrieli S Ghosh S Nieto-Castanon A Saygin Z Doehrmann O Chai X. Brain connectomics predict response to treatment in social anxiety disorder. Mol Psychiatry. (2016) 21:680. 10.1038/mp.2015.109 26260493

[49] Fan Y Shen D Gur RC Gur RE Davatzikos C COMPARE. classification of morphological patterns using adaptive regional elements. IEEE Trans Med Imaging. (2006) 26:93–105. 10.1109/TMI.2006.886812 17243588

[50] Haxby JV. Multivariate pattern analysis of fMRI: the early beginnings. Neuroimage. (2012) 62:852–5. 10.1016/j.neuroimage.2012.03.016 22425670 PMC3389290

[51] Tu CH Niddam DM Yeh TC Lirng JF Cheng CM Chou CC. Menstrual pain is associated with rapid structural alterations in the brain. Pain. (2013) 154:1718–24. 10.1016/j.pain.2013.05.022 23693160

[52] Wu TH Tu CH Chao HT Li WC Low I Chuang CY. Dynamic changes of functional pain connectome in women with primary dysmenorrhea. Sci Rep. (2016) 6:24543. 10.1038/srep24543 27089970 PMC4835697

[53] Shi Y Liu Z Zhang S Li Q Guo S Yang J. Brain network response to acupuncture stimuli in experimental acute low back pain: an fMRI study. Evid.Based Complemen Altern Med. (2015) 2015:210120. 10.1155/2015/210120 26161117 PMC4487721

[54] Lee J Eun S Kim J Lee JH Park K. Differential Influence of Acupuncture Somatosensory and Cognitive/Affective Components on Functional Brain Connectivity and Pain Reduction During Low Back Pain State. Front Neurosci. (2019) 13:1062. 10.3389/fnins.2019.01062 31636536 PMC6788296

[55] Chen X Spaeth RB Freeman SG Scarborough DM Hashmi JA Wey HY. The modulation effect of longitudinal acupuncture on resting state functional connectivity in knee osteoarthritis patients. Mol Pain. (2015) 11:67. 10.1186/s12990-015-0071-9 26511911 PMC4625557

[56] Zhang Y Zhang H Nierhaus T Pach D Witt CM Yi M. Default mode network as a neural substrate of acupuncture: evidence, challenges and strategy. Front Neurosci. (2019) 13:100. 10.3389/fnins.2019.00100 30804749 PMC6378290

[57] Dhond RP Yeh C Park K Kettner N Napadow V. Acupuncture modulates resting state connectivity in default and sensorimotor brain networks. Pain. (2008) 136:407–18. 10.1016/j.pain.2008.01.011 18337009 PMC2440647

[58] Keshavan MS Collin G Guimond S Kelly S Prasad KM Lizano P. Neuroimaging in Schizophrenia. Neuroimaging Clin N Am. (2020) 30:73–83. 10.1016/j.nic.2019.09.007 31759574 PMC7724147

[59] Janssen RJ Mourão-Miranda J Schnack HG. Making individual prognoses in psychiatry using neuroimaging and machine learning. Biol Psychiatry Cogn Neurosci Neuroimaging. (2018) 3:798–808. 10.1016/j.bpsc.2018.04.004 29789268

[60] Gao S Calhoun VD Sui J. Machine learning in major depression: From classification to treatment outcome prediction. CNS Neurosci Ther. (2018) 24:1037–52. 10.1111/cns.13048 30136381 PMC6324186

[61] Wu GR Wang X Baeken C. Baseline functional connectivity may predict placebo responses to accelerated rTMS treatment in major depression. Hum Brain Mapp. (2020) 41:632–9. 10.1002/hbm.24828 31633261 PMC7267925

[62] Leaver AM Wade B Vasavada M Hellemann G Joshi SH Espinoza R. Fronto-temporal connectivity predicts ECT outcome in major depression. Front Psychiatry. (2018) 9:92. 10.3389/fpsyt.2018.00092 29618992 PMC5871748

[63] Mithani K Mikhail M Morgan BR Wong S Weil AG Deschenes S. Connectomic profiling identifies responders to vagus nerve stimulation. Ann Neurol. (2019) 86:743–53. 10.1002/ana.25574 31393626

[64] Yang J Zeng F Feng Y Fang L Qin W Liu X. A PET-CT study on the specificity of acupoints through acupuncture treatment in migraine patients. BMC Complement Altern Med. (2012) 12:123. 10.1186/1472-6882-12-123 22894176 PMC3480944

[65] Yu S Ortiz A Gollub RL Wilson G Gerber J Park J. Acupuncture Treatment Modulates the Connectivity of Key Regions of the Descending Pain Modulation and Reward Systems in Patients with Chronic Low Back Pain. J Clin Med. (2020) 9:1719. 10.3390/jcm9061719 32503194 PMC7356178

